# Evaluation of a large-scale biomedical data annotation initiative

**DOI:** 10.1186/1471-2105-10-S9-S10

**Published:** 2009-09-17

**Authors:** Ronilda Lacson, Erik Pitzer, Christian Hinske, Pedro Galante, Lucila Ohno-Machado

**Affiliations:** 1 Decision Systems Group, Brigham & Women's Hospital, Harvard Medical School, Boston, MA, USA; 2 Upper Austria University of Applied Sciences, Hagenberg, Austria; 3 Ludwig Institute for Cancer Research, Sao Paolo Branch, Sao Paulo, Brazil

## Abstract

**Background:**

This study describes a large-scale manual re-annotation of data samples in the Gene Expression Omnibus (GEO), using variables and values derived from the National Cancer Institute thesaurus. A framework is described for creating an annotation scheme for various diseases that is flexible, comprehensive, and scalable. The annotation structure is evaluated by measuring coverage and agreement between annotators.

**Results:**

There were 12,500 samples annotated with approximately 30 variables, in each of six disease categories – breast cancer, colon cancer, inflammatory bowel disease (IBD), rheumatoid arthritis (RA), systemic lupus erythematosus (SLE), and Type 1 diabetes mellitus (DM). The annotators provided excellent variable coverage, with known values for over 98% of three critical variables: disease state, tissue, and sample type. There was 89% strict inter-annotator agreement and 92% agreement when using semantic and partial similarity measures.

**Conclusion:**

We show that it is possible to perform manual re-annotation of a large repository in a reliable manner.

## Background

Large repositories of gene expression data are currently available and serve as online resources for researchers, including the Gene Expression Omnibus (GEO), the Center for Information Biology Gene Expression Database (CIBEX), the European Bioinformatics Institute's ArrayExpress and the Stanford Tissue Microarray Database [1-4]. Repositories for gene expression data such as GEO allow for widespread distribution of gene expression measurements in order to: (1) validate experimental results, (2) enable progressive accumulation of data that may support, modify or further develop prior work, and (3) facilitate use of archived measurements to generate novel hypotheses that naturally develop from continuous updating of accumulated data. Although GEO contains a vast amount of measurements from numerous samples, the link between measurements and phenotypic characteristics of each individual sample, including the sample's disease and tissue type, is not readily accessible because they are encoded as free text. Furthermore, there are no standardized documentation rules, so phenotypic and/or protocol information resides in multiple documents and physical locations. Such information may be included as text describing the experiment or protocol, sample and sampling descriptions, or may be found only in the published journal article that may accompany the submission. In order to increase utility and improve ease of use of this resource, data should be readily available and easily comprehensible, not only for researchers, but also for automatic retrieval. In particular, the data have to contain sufficient detail to allow for appropriate combination of similar experimental subjects and protocols that may then collectively facilitate the verification, support, or development of new hypotheses.

Many centers have focused on re-annotating biomedical data with the goal of increasing utility for researchers. The promise of fast-paced annotation amid rapid accumulation of data has spurred great interest in progressive development of automated methods [4,5]. To date, manually annotated data is the de facto gold standard for most annotation efforts [4,5]. Therefore, it becomes critical to ensure that manually annotated data are accurately described and evaluated.

Several attempts directed specifically at annotation of gene expression data have been performed and remain the subject of ongoing work. In particular, GEO datasets (GDS) are being developed to systematically categorize statistically and biologically similar samples that were processed using a similar platform within a single study [6]. The process typically begins with a GEO series (GSE), defined as an experiment deposited into GEO that contains descriptions of the samples within the experiment, usually provided by the investigator. A GSE is then characterized into a data set. This phase is performed manually, with reviewers adjudicating whether or not experiments are comparable, which of them should belong in a dataset, and what axis differentiates samples from each other within a dataset. Some commonly used axes include the disease state and the cell line. Table 1a illustrates common descriptions that are given for samples within a GSE that correspond to various axes. There are 24 distinct axes that are currently in use. Each GDS, however, only utilizes a few axes, at the discretion of the curators. In addition, while the axes used to group samples are controlled, the values corresponding to these axes are typically provided as free text. The vocabulary used to describe the values within an axis is neither standardized nor controlled. To illustrate, *breast cancer *is entered as a value for a "disease state", whereas *breast tumor *is entered as a value for "cell line" in the sample excerpted in Table 1b. Moreover, the reference to *breast tumor *is ambiguous under "cell line" because this axis should specifically refer to breast cancer instead of tumor, given that these cell lines refer to models of neoplastic diseases.

**Table 1:** (a) taken from GDS showing three axes – "cell line," "disease state", and "stress" with corresponding values; (b) taken from GDS showing cell line descriptors.

**Type**	**Description**
**(a)**	

Cell line	HTB26
Cell line	HT29
Disease state	Breast cancer
Disease state	Colon cancer
Stress	Caspase inactivated
Stress	DNA fragmented
**(b)**	

Cell line	Breast tumor
Cell line	Colon tumor

It is not surprising, therefore, that re-annotating GEO and other large microarray data repositories is the focus of several groups. In particular, automatic text processing is being used to capture disease states corresponding to a given sample from GDS annotations. In a recently published article in which the objective was to identify disease and control samples within an experiment, the GDS subsets were analyzed using representative text phrases and algorithms for negation and lexical variation [5]. Although this algorithm was successful in identifying 62% of controls, the study was evaluated using only 200 samples, and it highlighted an urgent need for a methodical solution for annotating GEO using a controlled vocabulary. Another study performed re-annotation of the Stanford Tissue Microarray Database using the National Cancer Institute (NCI) thesaurus [4]. They were successful in representing annotations for 86% of the samples with 86% precision and 87% recall, but the study was evaluated using only 300 samples. While diagnosis remains as one of the most useful annotation points for a given experimental sample, there are many more categories of interest to investigators and users. For example, treatment interventions, sample demographics (e.g. age, gender, race), and various phenotypic information that affects gene expression. A re-annotation of these rapidly growing repositories has to take into account all these variables and the use of a controlled vocabulary for identifying sample variables and values.

We therefore describe a large-scale manual re-annotation of data samples in GEO, including variable fields derived from the NCI thesaurus and corresponding values that also utilize primarily controlled terminology [7]. The objective is to create an annotation scheme for various disease states that is flexible, comprehensive and scalable. We subsequently present a framework for evaluating the annotation structure by measuring coverage and agreement between annotators.

## Methods

Three sections below specifically: (1) enumerate the iterative process used for developing an annotation structure, (2) describe the annotation tool and the annotators' characteristics, and (3) describe the framework for evaluation.

An iterative process was designed for identifying the variables selected for annotation, as follows:

**1. Variable generation **– Human experts develop a list of variables for annotation. This procedure is based on guidelines and publications that are related to the disease category. Variables were then trimmed based on consensus among three physicians.

**2. Supervised domain annotation **– A trained annotator was instructed to start annotating the given variables under physician supervision. Whenever a variable deemed important was identified, it was listed for further deliberation. The process was then repeated – back to number (1) above, until no further variables were identified or the amount of samples for preliminary annotation was reached (i.e. 10% of the total samples for annotation within each domain).

**3. Unsupervised annotation **– A trained human annotator then performed unsupervised annotation independently, after receiving a standardized, written instruction protocol. Instructions were specifically developed for each disease category. Two human annotators were assigned to code each data sample. Randomized assignment between annotators was performed by disease category to minimize the occurrence of two coders being assigned to annotate the same disease category (and therefore the same samples) repeatedly.

**4. Disagreement and partial agreement identification **– After the human annotators finished coding their assigned experiments, the data was compiled and the assigned values were compared to measure agreement. The method to assess agreement is further described below.

**5. Re-annotation **– Finally, the samples containing values that were not in agreement initially were re-annotated and the correct annotation was determined by a majority vote. In the event of a three-way tie, one of the investigators performed a manual review and final adjudication.

**Figure 1:**
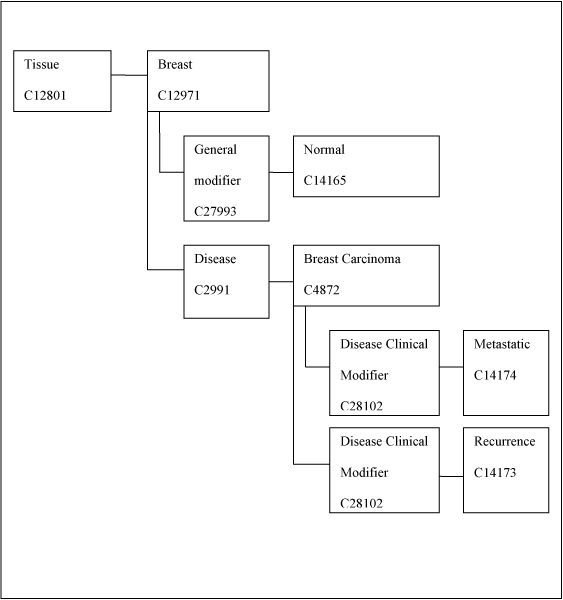
**Illustration of concepts derived from NCI thesaurus used for variables and values**.

To ensure consistency of terminology, the NCI thesaurus was utilized for the disease domains annotated, consistent with prior annotation initiatives [4,8]. This ensures that the concepts utilized all readily mapped to the Unified Medical Language System (UMLS) [9]. Therefore, scalability for using variables and values was preserved, which is valuable for future research initiatives. Figure 1 below shows a graphical illustration of the variable and values that were utilized to annotate breast cancer.

The variable "tissue" was assigned several different values, one of which was "breast." This assignment provided flexibility, allowing for addition of other tissue types, whenever the disease domain changes. There was also sufficient granularity to allow for actual interrogation(s) into the database for future hypothesis generation or validation. A full description of the web-based annotation tool and the quantity of samples annotated over time is described in a separate paper [10].

### Evaluation of annotations

There were a total of six annotators, including four senior biology students, one graduate student in the biological sciences field, and one physician. As noted previously, each sample had at least two annotators assigning values to variables. The annotation task was to provide phenotypic information for each data sample that was available in GEO for breast and colon cancer, IBD, DM, SLE, and RA. Thus, it was critical to obtain standardized values for most of the annotation variables to ensure that the annotations would be consistent. This entailed a review of data descriptions listed in various sources – the data sets (GDS), series information (GSE) and sample information (GSM). In addition, information was available in supplementary files and in published scientific articles, which are not in GEO. Manual review of all these data sources was necessary to obtain sufficient variable coverage. Coverage was defined as the percentage of non-'unknown' values that were assigned to a variable. Specifically, it can be represented as:

Coverage = X/Y, where X represents the number of variables with values that are not "unknown." Y represents the total number of variables that were annotated.

Table 2 illustrates how agreement was measured. Because our annotation tool limited the use of free text and confined most variables and values to predetermined concepts from the NCI Thesaurus, there was little distinction between strict similarity and semantic similarity. On the other hand, a review of prior annotation initiatives used semantic similarity, so we also utilized this index [5,11]. Finally, partial similarity measured the presence of text that has some degree of similarity to another [12]. In particular, whenever there is a variable that has a value "yes" or "no," with further specific detail corresponding to the variable, agreement on the binary value with a discrepancy in the specific detail would warrant a partial match. For example, partial agreement was assessed if an annotator provided a cell line name and another annotator left it blank, as long as both annotators agreed that the sample was from a cell line.

**Table 2:** Criteria for measuring agreement

**Agreement Type**	**Description**
Strict similarity	Exactly the same variable value between annotators.
Semantic similarity	There is lexical discordance, but the words match to the same concept. This subsumes hierarchical similarity.
Partial similarity	Partial agreement, some degree of discordance.

To validate the reliability of the annotation scheme, we computed the percentage of agreement between annotators, defined as the number of variables for which both annotators gave the same value, divided by the total number of variables that were annotated. We calculated percentage agreement for each level of similarity across all disease categories.

## Results

### Data description

A substantial fraction of GEO, including 45 platforms, 2,445 studies, and 58,432 samples were extracted into the analytical database. Among them, several disease categories are represented, but only 11,511 samples (19.7%) are included in various GDS subsets. Over a period of five weeks, 12,500 samples (21.4%) from a limited set of disease categories were annotated, as shown in Table 3. Many of these did not have annotations in GDS.

**Table 3:** Disease categories annotated from GEO

**Disease Category**	**# variables**
Breast Cancer	41
Colon Cancer	30
Inflammatory Bowel Disease (IBD)	30
Insulin Dependent Diabetes Mellitus (DM)	21
Rheumatoid Arthritis (RA)	19
Systemic Lupus Erythematosus (SLE)	32

In addition, for each disease category, a comprehensive and controlled set of phenotypic variables were provided, as shown in Table 4. For each disease category, between 19 and 41 variables were identified (see Table 3). To our knowledge, this constitutes the largest re-annotation initiative performed on gene expression data to date.

**Table 4:** Sample variables that are annotated for three disease categories – breast and colon cancer and rheumatoid arthritis

**Disease Category**	**Generic Variables**	**Disease-Specific Variables**
Breast cancer	Age Gender	ER/PR Past breast cancer Cancer Grade
		
Colon cancer		Duke staging Degree of differentiation
		
Rheumatoid arthritis		Cell type CD classification Rheumatoid factor

The next goal was to provide adequate coverage for as many variables that were identified. Table 5 shows the top 10 most commonly annotated variables and their coverage. As shown in Table 5, the sample tissue, cell line and disease states were most frequently annotated and were rarely "unknown". These were probably the most pertinent variables and likely the subject of most re-annotation initiatives. Therefore, it was critical that values for these variables were available and actually annotated.

**Table 5:** Coverage of the top ten variables

**Top Ten Variables**	**NCI Thesaurus ID**	**Coverage (%)**
Tissue	C12801	99.7
Cell line	C16403	99.5
Disease state	C2991	98.9
Sample type	C70713	98.0
Genetically modified	C16621+C42629	92.8
Treatment	C49236	76.2
Treatment type	C49236+C27993	71.5
Time series	C18235	67.2
Gender	C17357	59.9
Age	C25150	53.2

Inter-annotator agreement results are shown in Table 6. There is 89.3% strict agreement. There was a 1.7% difference between strict and semantic agreement in this study. Further improvement in agreement (1.2%) was observed when partial similarity was measured.

**Table 6:** Inter-annotator agreement

**Agreement Type**	**% Agreement**
Strict	89.3
Semantic	91.0
Semantic + Partia	l92.2

Overall, there was excellent inter-annotator agreement across multiple disease domains. Table 7 shows examples of the most common types of disagreements that we observed between annotators. Most commonly, one annotator labels a sample variable (e.g. treatment) as "unknown," while another annotator labels the same variable with the value "no" (e.g. no treatment).

**Table 7:** Disagreement between Annotators

**Variable**	**Disagreement**	
	
	Annotator 1	Annotator 2
Treatment type	unknown	no
Treatment	unknown	yes
Sample type	unknown	tumor
Stage	2	2a
TNM classification	T4b N2a M0	T4b N2a M3b
Family history	no	yes

## Discussion

Repositories for gene expression data such as GEO are expanding very rapidly [13]. However, the critical details necessary for understanding the experiments and sample information are encoded as free text and are not readily available for analysis. We described a large scale re-annotation performed on a substantial portion of the GEO consisting of 12,500 samples. Our large scale re-annotation was accomplished within a reasonable amount of time – completed within only five weeks. In addition, we were able to accomplish annotations of samples in great detail. The annotations used controlled terminology from the NCI thesaurus, with the advantage of allowing generalizability of the annotations for other research applications.

This study's re-annotation evaluation was performed on sample quantities that are two orders of magnitude higher than most prior reports [4,5,12]. A major contribution of this research effort includes the massive amount of well-annotated data, with substantial coverage for a large number of phenotypic information and with excellent accuracy, particularly at the semantic level.

We also described the methodology used for identifying relevant variables in each disease category. This iterative process is efficient and provided a mechanism for identifying relevant variables for domain categories. This technique provides a framework for inducing structure of a specific domain in an iterative and consultative manner. Excellent inter-annotator agreement confirmed that the annotation variables were robust and easily identifiable.

Finally, we provided a framework for measuring inter-annotator agreement. Apart from strict agreement measured using exact string matching between variable values, we defined and considered two other similarity categories that were known to be especially useful for annotations that relied heavily on free text. We showed an improvement in agreement using these more lenient similarity measures. The degree of improvement was mitigated by the very controlled terminology from the NCI Thesaurus that annotators utilized, and was augmented by the annotation tool. Several studies use semantic similarity as a measurement of agreement in annotation of microarray data [4,5]. Several other studies use partial agreement, especially when annotated text contains fragments that are not exactly similar [12,14]. Manual curation is usually the gold standard and determines whether terms that were used are semantically appropriate or not [15]. Our results show better strict, semantic, and partial agreement compared to most other re-annotation studies [12,16].

## Conclusion

Phenotypic annotations and data sample information are critically important for translational research. In particular, it is important to have good coverage for vital information, specific to clinical domain, as well as providing accurate annotations. We show that it is possible to perform manual re-annotation of a large repository in a reliable and efficient manner.

## Competing interests

The authors declare that they have no competing interests.

## Authors' contributions

All authors were involved in designing the study, and developing the annotation structure. Likewise, all authors were involved in the annotation system design and development of the user interface. After the initial pilot phase, RL, CH and LOM were involved in further variable identification and selection. CH was involved in performing some of the annotation in the pilot phase. RL was involved in supervising the entire annotation process and evaluating annotation quality. All authors contributed to preparation of this manuscript and read and approved the final version.
